# Orthopedic Provider Gender Preference Among Patients in an Orthopedic Surgery Residency Program of a Hispanic American Community

**DOI:** 10.1089/whr.2021.0126

**Published:** 2022-03-16

**Authors:** Elena Iguina-González, Gerardo Olivella, Andrea Ramos-Vicente, Andrés Fantauzzi, Ana Dávila, Danny Mangual, Norberto J. Torres-Lugo, Gladys Ramos, Norman Ramírez, Antonio Otero-López, Ariel Dávila-Parrilla

**Affiliations:** ^1^ Department of Orthopaedic Surgery, University of Puerto Rico, Medical Sciences Campus, San Juan, Puerto Rico.; ^2^ Department of Internal Medicine-Pediatrics, University of Puerto Rico, Medical Sciences Campus, San Juan, Puerto Rico.; ^3^ Department of Surgery, University of Puerto Rico, Medical Sciences Campus, San Juan, Puerto Rico.; ^4^ School of Medicine, University of Puerto Rico, Medical Sciences Campus, San Juan, Puerto Rico.; ^5^ Department of Pediatric Orthopaedic Surgery, Mayaguez Medical Center, Mayaguez, Puerto Rico.

**Keywords:** female orthopedic surgeons, diversity, Hispanic Americans, patient preferences

## Abstract

**Background:**

There are limited data regarding the gender preferences of Hispanic Americans when selecting their orthopedic surgeon. This study aimed to evaluate the gender preferences of Hispanic Americans when choosing a physician as their orthopedic provider.

**Materials and Methods:**

A cross-sectional survey was administered to all consecutive Hispanic American patients treated at the outpatient orthopedic clinics of a tertiary medical center in Puerto Rico between October 4, 2019 and March 4, 2020. Sociodemographic status and opinion of gender preference in orthopedic surgery were assessed and analyzed between female and male respondents.

**Results:**

A total of 628 surveys were completed. There were 343 (54.6%) females and 285 (45.4%) males with an average age of 51.0 ± 13.0 years. A significantly higher portion of female respondents was widowed (*p* = 0.01), had a higher educational level (*p* = 0.02), were unemployed (*p* = 0.01), and had a lower individual annual income salary (*p* = 0.04); when compared with males. Most of the respondents had no gender preference (91.1% = 572/628) for an orthopedic provider. Among those with a gender preference, 5.1% (32/628) preferred a male surgeon, and 3.8% (24/628) preferred a female surgeon. No significant difference was found between male and female respondents in the opinion of an orthopedic provider.

**Conclusions:**

This study illustrates that Hispanic Americans have no gender preference when choosing an orthopedic provider. Therefore, patient preference should not be considered a factor contributing to women's under-representation in our orthopedic surgery training program. Our findings may also assist future studies in search of other indications attributed to the under-representation of females in this field.

## Introduction

Orthopedic surgery has been known to be a male-dominated field.^1,2^ The promotion of gender diversity in orthopedic surgery has lagged behind other surgical specialties, showing the least progress in female involvement.^2,3^ Although more than half (51.0%) of first-year enrolled U.S. medical students are female, orthopedic surgery has the lowest representation of female residents (15.4%) when compared with all other surgical specialties.^4,5^ Likewise, the American Academy of Orthopedic Surgeons (AAOS) reports that only 7.6% of the orthopedic working community are females and only 17.8% work in academic positions.^6,7^

Understanding patients' views when selecting an orthopedic provider have been considered a possible factor contributing to female under-representation in this field.^1,8,9^ Previous studies, implemented in Europe and the U.S. mainland, have concluded that a patients' view may not play a role in the lack of female representation in orthopedic surgery.^1,8,9^ However, despite their conclusions on specific populations, there is no detailed analysis among Hispanic American patients.

Hispanic Americans are the largest minority group in the United States at 18% of the population.^10^ In this culture, males have been described with a dominant character toward women and children.^11,12^ Currently, there are no studies to our knowledge that analyze whether Hispanic American patients have a gender preference when selecting their orthopedic surgeon. This study aimed to evaluate the gender preferences of Hispanic Americans when selecting a physician as their orthopedic provider. We hypothesized that Hispanic American patients would choose a male physician over a female physician as their orthopedic provider.

## Materials and Methods

In this cross-sectional survey study, all consecutive Hispanic American patients waiting to be treated at the outpatient orthopedic clinics of a tertiary medical center in Puerto Rico from October 4, 2019 to March 4, 2020 were included. Each patient voluntarily completed a paper format questionnaire (Spanish or English version) documenting their sociodemographic status and opinion of physician gender role in orthopedic surgery.

To be eligible for the survey, the patient must have been 21 years old or older, visiting the orthopedic surgery outpatient clinics, able to read, comprehend and complete the survey, and be a new patient without a previous ongoing relationship with the clinical provider. Patients who did not meet the eligibility criteria were excluded from the study. All the participants were explained orally and verbally that the survey was entirely voluntary, anonymous, would not affect their treatment, had no compensation, and could withdraw from the study at any time. Faculty and resident physicians were informed about the study before its initiation and provided permission for the study.

The survey included 21 items distributed in two sections: sociodemographic (6 items) and opinion of physician gender role in orthopedic surgery (15 items). The sociodemographic section identified their age, gender, civil state, highest educational level achieved, employment status, and individual annual income salary. The opinion section evaluated the Hispanic Americans' views about gender physician base preference, capacity, and equality in orthopedic surgery. Most of the items were binary (yes or no) or 5-point Likert scale (comfortable, somewhat comfortable, indifferent, somewhat uncomfortable, or very uncomfortable) choice questions.^13^ The 5-point scale responses were then condensed to a 3-point scale (comfortable, indifferent, or uncomfortable) as described in previous studies.^14^

The survey responses were analyzed for differences between male and female respondents, using frequencies with percentages for categorical variables and means with standard deviations for continuous variables. An analysis of categorical and continuous variables was performed with a Fisher's exact test and Mann–Whitney *U* test, respectively. Statistical significance was determined at *p* < 0.05. The management and analyses were carried out with Microsoft Excel^®^ version 2016 (Microsoft Corp.) and SPSS version 24.0 (IBM Corp.). This study followed the Strengthening the Reporting of Observational Studies in Epidemiology (STROBE) reporting guideline for cross-sectional studies.^15^

This research was approved by the Institutional Review Board of a major teaching hospital in Puerto Rico.

## Results

There were 628 out of 647 patients (97.1% response rate) who visited our orthopedic clinics, agreed to participate, and completed the survey. Of the respondents, 343 patients (54.6%) were female, and 285 patients (45.4%) were male. Most of the respondents were between the ages of 26 and 65 years, with an average age of 51.0 ± 13.0 years (average age of males 50.4 ± 14.5 years; average age of females 51.4 ± 15.9 years).

All the participants identified themselves as Hispanic Americans. Eighty-nine percent of the participants had a high school diploma education or above. Three hundred one participants (47.9%) were employed. A significantly higher representation of female respondents was widowed (*p* = 0.01), had a higher educational level (*p* = 0.02), were unemployed (*p* = 0.01), and had a lower individual annual income salary (*p* = 0.04); when compared with males. The sociodemographic data of respondents are illustrated in Table 1.

**Table 1.** Sociodemographic Data of Male and Female Respondents (***N* = 628)**

Parameters	All respondents (*N* = 628), *n* (%)	Male respondents (*N* = 285), *n* (%)	Female respondents (*N* = 343), *n* (%)	*p*
1. Age				
21–25	35 (5.6)	19 (6.7)	16 (4.7)	0.298
26–50	245 (39.0)	111 (38.9)	134 (39.1)	1.000
51–65	239 (38.1)	115 (40.4)	124 (36.2)	0.284
>65	109 (17.4)	40 (14.0)	69 (20.1)	0.057
2. Gender				
Female	343 (54.6)	0 (0.0)	343 (100.0)	—
Male	285 (45.4)	285 (100.0)	0 (0.0)	—
3. Civil state				
Single	260 (41.4)	125 (43.9)	135 (39.4)	0.256
Married/partner	273 (43.5)	125 (43.9)	148 (43.1)	0.872
Divorced/separated	59 (9.4)	28 (9.8)	31 (9.0)	0.784
Widowed	36 (5.7)	7 (2.5)	29 (8.5)	0.002
4. Highest educational level				
Less than high school graduate	71 (11.3)	38 (13.4)	33 (9.6)	0.164
High school graduate	188 (29.9)	105 (36.8)	83 (24.2)	0.001
Some college or 2-year degree	87 (13.9)	32 (11.2)	55 (16.0)	0.104
4-Year college graduate	208 (33.1)	81 (28.4)	127 (37.0)	0.027
More than 4-year college degree	74 (11.8)	29 (10.2)	45 (13.1)	0.266
5. Employment status				
Unemployed	219 (34.9)	75 (26.3)	144 (42.0)	0.001
Retired	108 (17.2)	55 (19.3)	53 (15.5)	0.205
Employed	301 (47.9)	155 (54.4)	146 (42.6)	0.004
6. Annual income salary				
<$25,000	479 (76.3)	211 (74.0)	268 (78.1)	0.258
$25,001–$50,000	86 (13.7)	37 (13.0)	49 (14.3)	0.727
$50,001–$75,000	28 (4.5)	18 (6.3)	10 (2.9)	0.051
$75,001–$100,000	14 (2.2)	7 (2.5)	7 (2.0)	0.790
>$100,000	21 (3.3)	12 (4.2)	9 (2.6)	0.373

Overall, patients had a positive perception of females in orthopedic surgery. Most patients (91.1% = 572/628) did not have a gender preference when choosing their orthopedic surgeon. Among those with a gender preference, 5.1% (32/628) preferred a male surgeon, and 3.8% (24/628) preferred a female surgeon. No significant differences were found between male and female respondents toward the gender preference of an orthopedic provider (see Table 2).

**Table 2.** Hispanic's Perspective About Their Orthopedic Provider Stratified by Male and Female Respondents (***N* = 628)**

Parameters	All (*N* = 628), *n* (%)	Male (*n* = 285), *n* (%)	Female (*n* = 343), *n* (%)	*p*
7. Did you choose your orthopedic provider?				
Yes	224 (35.7)	93 (32.6)	131 (38.2)	0.156
8. Did you choose your orthopedic provider by referral?				
Yes	162 (25.8)	65 (22.8)	97 (28.3)	0.121
9. Did you choose your orthopedic provider because of their good reputation?				
Yes	33 (5.3)	16 (5.6)	17 (5.0)	0.723
10. Did you choose your orthopedic provider because he was a man?				
Yes	2 (0.3)	2 (0.7)	0 (0.0)	0.206
11. Did you choose your orthopedic provider because she was a woman?				
Yes	1 (0.2)	0 (0.0)	1 (0.3)	1.000
12. Do you prefer your orthopedic provider to be male or woman?				
Male	32 (5.1)	14 (4.9)	18 (5.2)	0.467
Woman	24 (3.8)	8 (2.8)	16 (4.7)	0.297
No preference	572 (91.1)	263 (92.3)	309 (90.1)	0.399
13. Do you think orthopedics is a suitable profession for both women and men?				
Yes	618 (98.4)	280 (98.2)	338 (98.5)	0.762
14. Do you feel that a man does a better job than a woman in orthopedics?				
Yes	2 (0.3)	0 (0.0)	2 (0.6)	0.504
15. Do you think that orthopedics is not suitable for women because it requires greater physical strength?				
Yes	8 (1.3)	5 (1.8)	3 (0.9)	0.479
16. Do you think that orthopedics is not suitable for women because they should dedicate their time to take care for their children?				
Yes	2 (0.3)	0 (0.0)	2 (0.6)	0.504
17. Do you think that orthopedics is not suitable for women because women are more delicate?				
Yes	0 (0.0)	2 (0.7)	0 (0.0)	0.206
How much would you agree with the following premises?				
18. Feels comfortable being cared by a woman as orthopedic provider				
Uncomfortable	41 (6.5)	19 (6.7)	22 (6.4)	1.000
Indifferent	145 (23.1)	55 (19.3)	90 (26.2)	0.051
Comfortable	442 (70.4)	211 (74.0)	231 (67.3)	0.081
19. Women have equal intellectual capacity as men to successfully work as an orthopedic provider				
Uncomfortable	31 (5.0)	12 (4.2)	19 (5.6)	0.466
Indifferent	24 (3.8)	15 (5.3)	9 (2.6)	0.097
Comfortable	573 (91.2)	258 (90.5)	315 (91.8)	0.574
20. Women would do as good as a job as a male in orthopedic surgery				
Uncomfortable	26 (4.1)	12 (4.3)	14 (4.1)	1.000
Indifferent	21 (3.3)	14 (4.9)	7 (2.0)	0.072
Comfortable	581 (92.5)	259 (90.8)	322 (93.9)	0.172
21. Should women receive equal payment?				
Uncomfortable	25 (4.0)	11 (3.9)	14 (4.1)	1.000
Indifferent	17 (2.7)	12 (4.2)	5 (1.5)	0.051
Comfortable	586 (93.3)	262 (91.9)	324 (94.4)	0.261

Among the 32 patients who preferred a male surgeon, 56.3% were female, and 43.8% were male. The majority were >51 years of age, married, unemployed, and had a low individual annual income salary. Most of the respondents who chose a male as their orthopedic provider were mainly due to their referral from another physician and a good reputation. Interestingly, four of these respondents stated that orthopedic was not suitable for females due to the requirement of greater physical strength.

In contrast, 24 patients preferred a female as their orthopedic provider (66.7% female and 33.3% male). Most of these respondents had similar sociodemographic profiles as those with a male preference, except for being mostly single. Furthermore, referral and good reputation were the most common reasons reported among those participants who chose a female as their orthopedic provider.

## Discussion

Orthopedic surgery is one of the least diverse medical specialties, despite increasing awareness of this issue.^3,5^ Even though there is a higher representation of females entering U.S. medical schools, the percentage of females in orthopedic surgery residencies has remained between 13% and 15% in the past decade.^3,4^ Furthermore, a recent study evidenced that orthopedic surgery had the slowest growth of female residents' proportion, requiring 138 years to reach gender equality in the field.^16^ The lack of interest in orthopedic surgery by females during medical school and the under-representation in residency perpetuates the lower percentage of women in the working community and academic field.^6,7^

Variables such as implicit gender bias, work-life balance workload, physical strength, low exposure in the field before and during medical school, lack of female mentors, low senior faculty acceptance, and patient's gender preferences when choosing an orthopedic provider; have been considered possible contributors to female under-representation in orthopedic surgery training programs.^1,4,6,8,9,17^

Three studies have analyzed whether patients have a gender preference when selecting their orthopedic surgeon.^1,8,9^ In 2009, Bucknall and Pynsent performed a 182-patient survey in England, where 82.0% expressed no gender preference for their orthopedic provider.^9^ A few years later, Abghari et al. conducted a 500-patient study in New York City, showing that their patients did not emphasize gender when choosing their orthopedic provider.^8^ Finally, Dineen et al. evaluated the views of 191 patients in North Carolina, showing an indifference of gender preference when choosing an orthopedic provider.^1^

In our academic medical center, the Accreditation Council for Graduate Medical Education (ACGME) orthopedic surgery residency has graduated 151 residents since 1967.^18,19^ Out of these graduates, only eight have been female, and only one is currently involved in an academic setting.^18,19^ Despite the female under-representation in our institution, most Hispanic American patients in this study had no gender preference when choosing their orthopedic provider in a traditionally “male-dominated” field. Therefore, our findings validate the conclusions of previous studies, emphasizing that Hispanic Americans' views toward female orthopedic surgeons should not be considered a factor contributing to the under-representation of women in this field.^1^

The quest to promote gender diversity in orthopedic surgery must continue. Other variables have been proposed as possible explanations for the lack of diversity and female under-representation in orthopedic surgery. The goal of diversity among social sciences is defined when 30% of the population becomes incorporated and represented in the institutional culture.^20,21^ In 2020, Van Heest stated that the main problem to achieve gender diversity in orthopedic surgery is that female medical students do not choose orthopedic surgery as a career.^20^ They also illustrated that the 30% diversity goal of female representation in orthopedic surgery residencies could be achieved by 2072.^20^

Despite this, recent studies have shown that current pipeline programs such as the Perry Initiative and Nth Dimensions are starting to become effective in recruiting female medical students into the profession of orthopedic surgery.^20,22,23^ Although our study was limited to patients' views, we agree that the accessibility of these programs at every U.S. medical school could increase the interest of females in orthopedic surgery.^20^ Future studies related to the views of medical students and residents in orthopedic surgery among a Hispanic American population could also help understand the necessities and required tools needed to increase the diversity in this field.

There are some limitations to our study. First, our survey has not been used nor validated by any other study. Second, patients may have responded to the survey thinking it would affect their treatment and predisposed to a Hawthorne effect, choosing a socially acceptable answer that may not follow their true preferences.^1^ Third, we evaluate individual income as a sociodemographic item; however, this could be misleading as the house partner could contribute a significant portion of the household income and be misleading in terms of the assumed socioeconomic status based solely on the individual income. Finally, we could not evaluate the patient's view based on orthopedic subspecialties.

## Conclusions

The authors of this study conclude that Hispanic Americans' views are not likely to contribute to the gender disparity in orthopedic surgery. Orthopedic surgery continues to lag behind other surgical specialties in female representation. Future studies should evaluate the impact of gender bias from a medical student and residency perspective among the Hispanic American community.

## Data Availability

All data generated or analyzed during this study are included in this published article.
